# Measuring Motivations to Eat Palatable Foods: Adaptation and Psychometric Properties of the Italian Version of the Palatable Eating Motives Scale (PEMS-IT)

**DOI:** 10.3390/healthcare12050574

**Published:** 2024-02-29

**Authors:** Giada Pietrabissa, Gianluca Castelnuovo, Michelle Semonella, Stefania Mannarini, Alessandro Alberto Rossi

**Affiliations:** 1 Department of Psychology, Catholic University of Milan, 20123 Milan, Italy; gianluca.castelnuovo@unicatt.it; 2 Clinical Psychology Research Laboratory, IRCCS Istituto Auxologico Italiano, 20149 Milan, Italy; 3 Department of Psychology, Bar-Ilan University, Ramat Gan 5290002, Israel; michelle.semonella@biu.ac.il; 4 Department of Philosophy, Sociology, Education, and Applied Psychology, Section of Applied Psychology, University of Padova, 35131 Padova, Italy; stefania.mannarini@unipd.it (S.M.); a.rossi@unipd.it (A.A.R.); 5 Interdepartmental Center for Family Research, University of Padova, 35131 Padova, Italy

**Keywords:** food addiction, palatable foods, eating disorders, motivation, eating behaviors

## Abstract

Background: Gaining knowledge of the various reasons behind people’s consumption of highly processed foods has the potential to enhance obesity prevention initiatives and open avenues to tailor treatment approaches for obesity and binge eating at a more personalized level. This contribution aimed to test the psychometric properties and factor structure of the Palatable Eating Motives Scale (PEMS-IT) in a community sample of Italian adults. Methods: A confirmatory factor analysis was performed to test the factor structure of the Italian version of the PEMS (PEMS-IT) on a total of 616 respondents. Furthermore, the reliability and convergent validity analysis of the tool were evaluated. Results: The analysis confirmed the four-factor structure of PEMS-IT [(YBχ^2^ (164) = 537.901; *p* < 0.001, the CFI = 0.918, RMSEA = 0.072; 90%CI [0.065–0.078]; *p*(RMSEA < 0.05) < 0.001, and SRMR = 0.080] and satisfactory reliability on its subscales (Cronbach’s α: 0.745–0.917). Positive correlations were also found with food addiction and binge-eating symptoms, compulsive eating behavior, and uncontrolled and emotional eating. Conclusions: The PEMS-IT appears to be an instrument with promising psychometric properties and potential applications in clinical settings. However, it also has some limitations, and future studies could focus on improving the semantic content of the elements to increase the overall utility and precision of the instrument.

## 1. Introduction

Although there has been progress in understanding the environmental, genetic, and physiological factors that contribute to obesity, the global prevalence of this chronic disease remains elevated and is expected to increase [1,2]—as the challenge of maintaining weight loss is common even after initial success [3,4,5,6,7].

One potential factor contributing to the increase in overweight and obesity, as well as to the development of binge-eating disorders (BEDs), is the proliferation of highly processed foods (HPFs) [8]. These foods are typically characterized by high levels of fat, sugar, and salt, coupled with small amounts of vitamins, minerals, and fiber. Examples of highly processed foods are fizzy drinks, packaged snacks, cakes, and certain types of ready-to-eat foods. In addition, the abundance of food additives, including artificial colors, flavors, or preservatives, makes HPFs hyperpalatable and attractive.

Consumption of foods considered palatable is notoriously driven by pleasure rather than hunger or metabolic need [9,10]. Eating too much of them causes a profound state of reward hyposensitivity, similar to that of drug abuse, that can lead to the development of compulsive-like eating [11]. Therefore, high-carbohydrate and high-fat foods could be considered palatable. They are widely available to adults and children, both in terms of convenience and economic cost [12].

But people may differ in the reasons or motivations behind the consumption of hyperpalatable processed foods [13].

Some people may indulge in tasty foods for conventional or adaptive reasons, such as to celebrate an occasion, while others may do so for less adaptive reasons, including coping with negative emotions and stress.

Understanding what drives the consumption of hyper-processed palatable foods in the community could inform obesity prevention efforts and pave the way for more personalized treatment strategies for obesity and binge eating [14]. Furthermore, it could contribute to efforts to improve the nutritional quality of individuals’ diets [15].

A motivational model that has robust evidence supporting its validity in the context of alcohol use and has recently been extended to the consumption of hyper-processed palatable foods could offer a unified framework for understanding various motives that might drive hedonic eating [16].

Indeed, evidence suggests the rewarding effect of both alcohol and HPFs [17,18] and that energy overconsumption may be driven by the same motives across behaviors.

Among young adults, alcohol consumption for reward enhancement and social reasons is positively correlated with the level of alcohol consumption, while drinking to cope with negative emotions is directly associated with alcohol-related problems [19]. Similarly, eating to cope with negative emotions is related to binge-eating tendencies in studies conducted on college students [20,21] and motives such as reward enhancement, social reinforcement, and conformity also demonstrate significant associations with binge eating [17].

Hyper-processed palatable foods constitute the vast majority of energy intake during binge-eating episodes, and higher scores of hyper-processed food consumption are strongly correlated with increased chances of developing BEDs [22]. As for BEDs, food addiction is linked to greater reward sensitivity, greater food cravings, and hyper-responsiveness to HPFs [23,24,25].

Therefore, to deepen the knowledge of the motivations for consuming hyper-processed palatable foods, Burgess and colleagues (2014) created the Palatable Eating Motives Scale (PEMS) [17], a self-report scale derived from the revised Drinking Motives Questionnaire (DMQ-R) [26,27].

This questionnaire categorizes four reasons for eating palatable foods and beverages when not driven by hunger: coping, reward enhancement, social, and conformity motives.

Motives for coping encompass items that pertain to the consumption of palatable foods or drinks as a means of addressing negative emotions such as anxiety, sadness, frustration, or anger. The reward enhancement motive involves questions about the consumption of delicious foods or beverages to amplify positive experiences, emotions, or inherent satisfactory qualities that are not related to social contexts. For example, this may include eating certain foods that bring pleasure or contribute to personal happiness.

Social motives include inquiries about the consumption of palatable foods or beverages for social purposes, such as enjoying them at a party or participating in eating behaviors to socialize more with friends. On the other hand, the conformity motive involves questions about consuming tasty foods in response to external pressures, such as conforming to social expectations within a group of friends or adopting eating behaviors to avoid exclusion.

Since the PEMS is currently unavailable for implementation within the Italian population, this study aimed to adapt and test the psychometric properties of the Italian version of the PEMS (PEMS-IT) in a sample of Italian respondents from the general population and to examine its factor structure.

## 2. Materials and Methods

This research used a cross-sectional research study design to investigate the factor structure and psychometric properties of PEMS-IT among a sample of Italian adults from the community.

### 2.1. Translation and Cultural Adaptation

Translation and cross-cultural adaptation were carried out according to the cross-cultural adaptation guidelines of self-report measures proposed by Beaton et al. (2000), which included the following steps: 1. independent translations; 2. synthesis of these translations; 3. back-translations; 4. expert committee evaluation; and 5. pre-test of the pre-final version [28]. All translators involved in the process were bilingual and had a special professional background in psychology or other relevant areas.

- Step 1: Translation

First, the PEMS was translated from English into Italian by two natural multilingual translators from Italy. Along with clear directions, each translator independently translated the original content and response options. Neither the conceptual content of the questionnaire nor its concepts were known to either of the translators.

- Step 2: Synthesis of the translations

Subsequently, a synthesis of both translations was produced through discussion between the translators until a consensus was reached. Thus, a theoretically equivalent translation of the original questionnaire was obtained.

- Step 3: Back-translation

This reconciled version was then provided to a professional back-translator who was also bilingual and blind to the original English versions of the tool. The back-translated questionnaire was further compared with the original to detect any misinterpretation or imprecisions in the translation process.

- Step 4: Expert committee evaluation

Then, both the translation synthesis and the back-translated version of the PEMS were discussed between all translators and professionals involved in this study. These comprised two health professionals (psychotherapists) with many years of experience in the field of obesity and eating disorder treatment (authors G.C. and G.P.) and an expert in the validation of psychiatric questionnaires (author A.A.R.). Discussion and feedback were recorded and considered in the harmonization process. Once a consensus was reached on the identified differences in the proceeding, the PEMS-IT was proofread for grammar, spelling, and content errors, generating a pre-final version of the tool.

- Step 5: Pre-test of the pre-final version

Finally, the resulting version of the PEMS-IT was administered to a representative sample of 15 persons from the general population to assess the comprehensibility of the items. No further adjustments were made. The final version of the PEMS-IT is reported in Supplementary Materials S1.

### 2.2. Sample Size Calculation

The size of the sample was chosen a priori, and the *‘n*:*q’* criterion, which denotes the relationship between individuals and parameters, was used [29]. A requirement of 10 participants per parameter (=46) was established, culminating in a sample size of no fewer than 460 participants being determined.

### 2.3. Procedure

Based on the methodology employed in prior research [30,31,32,33], an online survey was conducted using Qualtrics software//February 2023 and distributed using the snowball sampling method on social networks (e.g., Facebook, Twitter, and Instagram). The recruitment materials provided detailed information on eligibility criteria and additional specifics to ensure that participants could make informed decisions, including assurances of anonymity for their responses. Those who agreed to participate completed the survey online.

Each participant was requested to provide sociodemographic (i.e., sex and age) and clinical information (weight and height, utilized for body mass index (BMI) calculation). Together with PEMS-IT, respondents were also asked to complete the Italian version of the following self-report questionnaires: the modified version of the Yale Food Addiction Scale 2.0 (mYFAS 2.0), the Binge Eating Scale (BES), the Measure of Eating Compulsivity 10-Italian version (MEC10-IT), the Three Factor Eating Questionnaire-18 (TFEQ-R-18), and the Repetitive Eating Questionnaire (Rep(Eat)-Q).

Ethical approval for the study was obtained from the Ethics Committee of the I.R.C.C.S. Istituto Auxologico Italiano, under protocol number 2020_02_18_04. All procedures adhered to the ethical standards set by the institutional and/or national research committee and were in accordance with the Declaration of Helsinki of 1964 and its subsequent amendments or equivalent ethical standards.

### 2.4. Participants

The inclusion criteria for the study participants were (A) being a native Italian speaker; (B) being 18 years or older; and (C) providing online consent to participate. Individuals were excluded if they (A) had visual and/or cognitive impairments that hindered the completion of the survey and (B) failed to respond to all the survey items.

The sample was made up of 616 respondents from the general population. The sample consisted of 474 women (76.9%) and 142 men (23.1%), between 18 and 84 years old (mean = 29.21, SD = 11.69) and with a BMI ranging from 14.84 to 55.74 kg/m^2^ (mean = 22.17, SD = 4.22, median = 21.30, skewness = 2.58, kurtosis = 11.84). More specifically, most of the sample was of normal weight (BMI of 18.5 to 24.9 kg/m^2^; *n* = 443; 71.9%), followed by underweight (BMI of 16 to 18.4 kg/m^2^; *n* = 70; 11.4%) and overweight (BMI of 25 to 29.9 kg/m^2^; *n* = 70; 11.4). The remaining part of the sample included class I obesity (BMI of 30 to 34.9 kg/m^2^; *n* = 13; 2.1%), class II obesity (BMI of 35 to 39.9 kg/m^2^; *n* = 9; 1.5%), class III obesity (BMI ≥ 40 kg/m^2^; *n* = 6; 1%), and severely underweight (BMI < 16 kg/m^2^; *n* = 5; 0.8%) [34].

### 2.5. Measures

The Palatable Eating Motives Scale (PEMS) [17] consists of 20 items answered on a 5-point Likert scale ranging from 1 that measures motivations for eating a variety of palatable foods and beverages across four main dimensions: social motives (Items 3, 5, 11, 14, 16); coping motives (Items 4, 6, 15, 17); reward enhancement motives (Items 7, 9, 10, 13, 18); and conformity motives (Items 2, 8, 12, 19, 20). Scores are obtained by the sum of these items.

The Modified Yale Food Addiction Scale 2.0 (mYFAS2.0) [30,35] comprises 11 diagnostic items from the original YFAS 2.0, along with two questions that assess impairment/distress. The scale retains the key characteristics of the original YFAS 2.0, including two scoring options (symptom count and diagnostic scores) and a diagnostic continuum indicating severity. All items are evaluated on an eight-point Likert scale, ranging from 0 (never) to 7 (every day). In the present sample, the internal consistency of mYFAS2.0 was KR20 = 0.852.

The Binge Eating Scale (BES) [36] consists of 16 items designed to assess prominent behavioral aspects (such as rapid eating or consuming large amounts of food) and affective/cognitive symptoms (such as guilt, a sense of being out of control, or an inability to stop eating) associated with episodes of binge eating. Each item presents 3 to 4 statements representing varying degrees of severity for the measured characteristics. Participants are asked to choose the statement that captures their personal experience. In the present study, BES showed acceptable internal consistency, with Cronbach’s alpha equal to 0.908.

The Italian version of the Measure of Eating Compulsivity (MEC10-IT) [32] is a brief, feasible, solid, and extremely reliable tool consisting of 10 items answered on a 5-point Likert-type scale ranging from 0 (very untrue) to 4 (very true) aimed at measuring compulsive eating behaviors and binge eating behaviors. High scores correspond to a high degree of eating compulsivity. The internal consistency of MEC10-IT in the present study was equal to 0.923.

The Three Factor Eating Questionnaire Revised–18 (IT-TFEQ-R-18) [37] is a reliable, solid, and psychometrically sound questionnaire that consists of 18 items measured on a 4-point Likert scale ranging from 1 (definitely false) to 4 (definitely true) designed to assess three main cognitive and behavioral domains of eating disorders: cognitive restraint (CR-6 items), uncontrolled eating (UE-9 items), and emotional eating (EE-3 items). High scores reflect a higher level of each dimension. In this study, the internal consistency of the IT-TFEQ-R-18 scales was 0.818 for the CR scale, 0.872 for the UE scale, and 0.884 for the EE scale.

The Repetitive Eating Questionnaire (Rep(Eat)-Q) [38] is a reliable, promising, and psychometrically based questionnaire that aims to measure a peculiar form of food addiction: grazing. It comprises 12 items answered on a 7-point Likert scale ranging from 0 (never) to 6 (every day) measuring the frequency of the attitudinal and behavioral characteristics of grazing during the past 28 days. It is made up of two dimensions: compulsive grazing (CG) and repetitive eating (RE), and a total score. The internal consistency of the Rep(Eat)-Q was equal to 0.910 for the total score, 0.887 for the RE scale, and 0.857 for the CG scale.

### 2.6. Statistical Analysis

Statistical analyses were performed with the R software—version 4.3.2 [39,40] and the following packages: lavaan [41,42], psych [43], psychTools [44], and tidyverse [45]. Graphical representations were made with the semPlot package [46].

**Figure 1:**
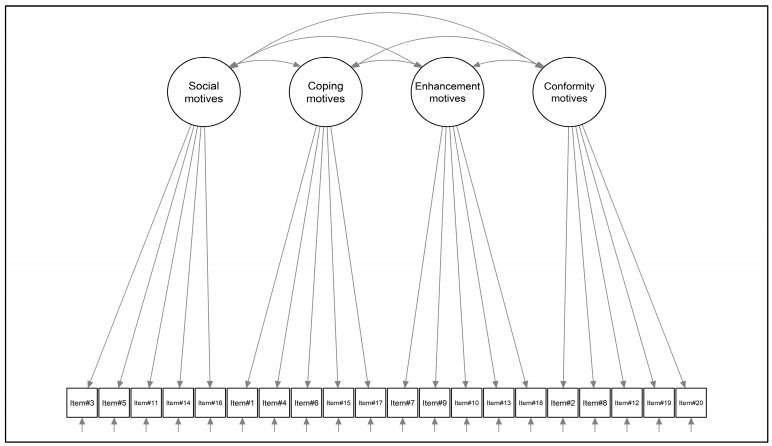
Structural model of the PEMS-IT.

According to its original validation study [17], a first-order model was specified comprising four correlated factors (Figure 1). Since the original validation study did not assume a ‘general factor’ (total score), no alternative models were tested. The MLR estimator (namely, robust maximum likelihood) was used to evaluate the factor structure of the PEMS-IT [47,48]—as some items were not normally distributed. Model fit was assessed using (A) the Yuan–Bentler Chi-square statistic (YBχ^2^), (B) the Root-Mean Square Error of Approximation (RMSEA), (C) the Comparative Fit Index (CFI), and (D) the Standardized Root Mean Residual (SRMR) [29,47,48]. To evaluate the goodness of fit, the following cut-off criteria were used: (A) statistical non-significance of the YBχ^2^, (B) an RMSEA less than 0.08, (C) a CFI higher than 0.95, and (D) an SRMR lower than 0.08 [29,49,50].

Once the factor structure of PEMS-IT was tested, its internal consistency was evaluated with Cronbach’s alpha (α) and McDonald’s omega (ω) [51]; for categorical/dichotomous items, the Kuder–Richardson20 (KR20) coefficient was used. Additionally, adjusted item–total correlation was calculated [52,53].

Convergence validity was performed using the Pearson correlation coefficient [52], with interpretations guided by Cohen’s standards: *r* < 0.10, negligible; *r* ranging from 0.10 to 0.30, minimal; *r* from 0.30 to 0.50, moderate; and *r* > 0.50, substantial [54].

## 3. Results

### 3.1. Structural Validity

The four-factor model (Figure 1) showed a good fit to the data: YBχ^2^ (164) = 537.901; *p* < 0.001, the CFI = 0.918, RMSEA = 0.072; 90%CI [0.065–0.078]; *p*(RMSEA < 0.05) < 0.001, and SRMR = 0.080. Social motive was weakly correlated with coping strategies (ϕ = 0.227), and moderately correlated with both reward enhancement (ϕ = 0.489) and conformity (ϕ = 0.541) motives. In addition, coping strategies were moderately correlated with reward enhancement motives (ϕ = 0.447) and weakly correlated with conformity motives (ϕ = 0.118). Last, reward enhancement motives did not correlate with conformity (ϕ = 0.045). The standardized factor loadings ranged from 0.317 (item#10; conformity) to 0.921 (item#4; coping). All statistics are shown in Table 1 below.

### 3.2. Internal Consistency

Internal consistency analysis revealed satisfactory results. Indeed, for the social motives scale, McDonald’s ω was equal to 0.845 and Cronbach’s α was equal to 0.840. For the coping motive scale, McDonald’s ω was equal to 0.922 and Cronbach’s α was equal to 0.917. In addition, for the reward enhancement motive scale, McDonald’s ω was equal to 0.844 and Cronbach’s α was equal to 0.804. Last, for the social motive scale, McDonald’s ω was equal to 0.749 and Cronbach’s α was equal to 0.745.

### 3.3. Convergent Validity

As shown in Table 2, small-to-moderate correlations were found between the PEMS-IT scales. Furthermore, considering the social motive scale, moderate associations were found with the PEMS-IT conformity motive scale (*r* = 0.546, *p* < 0.001) and the PEMS-IT reward enhancement motive scale (*r* = 0.447, *p* < 0.001). Still, only a few (small) correlations were found with other selected convergent measures. Indeed, small associations were found between the total score (*r* = 0.153, *p* < 0.001) and the Rep(Eat)-Q CG scale (*r* = 0.172, *p* < 0.001).

Regarding the coping motives scale, a moderate association was found with the PEMS-IT reward enhancement motive scale (*r* = 0.436, *p* < 0.001). Furthermore, moderate-to-large correlations were found with other convergent measures, particularly with scales that refer to excessive food intake. Indeed, moderate associations were found between the dimensions of the PEMS-IT coping motives and the mYFAS2.0 symptom count of mYFAS2.0 (*r* = 0.426, *p* < 0.001), the total score of BES (*r* = 0.587, *p* < 0.001), the total score of MEC10-IT (*r* = 0.554, *p* < 0.001), the TFEQ-R-18-uncontrolled eating scale (*r* = 0.498, *p* < 0.001), and the Rep(Eat)-Q total score (*r* = 0.528, *p* < 0.001). Furthermore, a large correlation was found with the TFEQ-R-18-emotional eating scale (*r* = 0.772, *p* < 0.001).

Taking into account the reward enhancement motive scale, small associations were found with other convergent measures: the highest association was with the TFEQ-R-18 uncontrolled eating scale (*r* = 0.343, *p* < 0.001), followed by the Rep(Eat)-Q total score (*r* = 0.282, *p* < 0.001) and the MEC10-IT total score (*r* = 0.248, *p* < 0.001).

Finally, small associations were found between the conformity motive scale and other convergent measures: the highest association was with the Rep(Eat)-Q CG scale (*r* = 0.167, *p* < 0.001), followed by the BES total score (*r* = 0.163, *p* < 0.001) and the TFEQ-R-18-emotional eating scale (*r* = 0.157, *p* < 0.001).

## 4. Discussion

Highly palatable foods play a relevant role in obesity and binge eating. A better understanding of whether individuals eat palatable foods primarily to cope, enhance reward, be social, or conform, measured by the PEMS, would inform the development of personalized treatment strategies for people suffering from eating and weight problems. For this reason, and since the tool is not yet available for use in Italy, the present contribution aimed to explore the psychometric proprieties of the PEMS and to test its factor structure in a sample of Italian adults from the general population.

The results of the confirmatory factor analysis (CFA) supported the structural validity of the PEMS-IT by confirming a four-factor structure: social, coping, reward enhancement, and conformity motives. Indeed, each of the 20 items loaded onto the theoretically hypothesized dimension with good fit indexes—thus demonstrating their successful portrayal of the underlying constructs [29,47].

Furthermore, considering the psychometric characteristics of the tool, reliability analyses revealed satisfactory results on all subscales.

Convergence validity analyses also showed small-to-large associations between the PEMS-IT subscales, as well as between the PEMS-IT subscales with other relevant measures. In particular, moderate-to-large correlations were found between the dimension of coping motives of the PEMS-IT and measures of excessive food intake, including the mYFAS2.0 symptom count (*r* = 0.426, *p* < 0.001), the BES score (*r* = 0.587, *p* < 0.001), the total MEC10-IT total score (*r* = 0.554, *p* < 0.001), the TFEQ-R-18-uncontrolled eating scale (*r* = 0.498, *p* < 0.001) and the emotional eating scale (*r* = 0.772, *p* < 0.001), and the Rep(Eat)-Q total score (*r* = 0.528, *p* < 0.001).

This suggests a tendency to eat HPFs in response to emotional urges. In fact, several systematic reviews and meta-analyses conclude that emotional states significantly impact individual diet choices and can result in the development of disordered eating behaviors and weight problems [55,56,57,58,59,60,61]. In particular, when individuals are under stress, a preference for highly palatable “snack-type” foods over “meal-type” foods has been observed. For example, Baumeister et al. (2002) noted that emotional eating tends to manifest more prominently in snacking behaviors [62]. Furthermore, a study by Boggiano et al. (2017) employing a sample of university students revealed significant positive correlations between the dimensions and the three subscales of the Emotional Eating Scale (EES) [63], namely anger/frustration, anxiety, and depression [21]. In particular, the PEMS coping and reward enhancement subscales (internal motives) correlated with higher BMI and were associated with increased eating concerns and emotion-triggered eating in both sexes, along with binge eating in females [8].

However, the results of this study should be interpreted in light of a few limitations. First, the cross-sectional nature of the sample and its testing only in a community sample, respectively, limit the evaluation of the temporal stability of the PEMS-IT and its external validity. Future research should evaluate the test–retest reliability of the tool and its measurement invariance between non-clinical and clinical samples. Another weakness of this research is that a limited number of emotional eating scales, such as the EES [63], were used to test convergence and incremental validity. Another limitation of this study was the lack of investigation into the divergent validity of the instrument. Future studies should consider evaluating this aspect using modern statistical approaches [64]. Furthermore, from a psychometric point of view, it is important to note that some items (Item#2 and Item#10), distributed on all questionnaire scales, exhibit factor loadings below 0.5. This suggests that although the model fits the data well (fit indices are good), these items do not optimally saturate the latent construct they are measuring. Future studies should consider developing a new version of the instrument by retaining the best items and replacing those that less accurately reflect the investigated construct. Furthermore, another limitation of this study is that the sample is mainly composed of women and that the age and BMI of the respondents vary a great deal, and this could impact the metabolic regulation and eating habits of the individuals. Future research should test possible explanatory models of motives that drive eating behaviors by considering the variables as possible intervening factors.

Despite the constraints mentioned above, this study marks the initial effort to assess the psychometric qualities of PEMS-IT within the broader population of Italy, supporting the validity and reliability of the tool. Importantly, these conclusions are based on robust statistical measures widely recognized throughout the world. Consequently, PEMS-IT emerges as a reliable option for clinical and research applications, as it proficiently detects the primary motivations for eating HPFs that are not related to hunger with precision and efficiency.

## 5. Conclusions

PEMS-IT is a useful measure for a rapid assessment of the baseline reasons for HPF consumption, which contribute greatly to obesity and eating-related disorders and influence individuals’ ability to adhere to weight reduction and rehabilitation programs [8]. In the present study, PEMS-IT shows promise for clinical applications; a thorough understanding of its psychometric properties, limitations, and potential areas for improvement is crucial to maximize its utility and precision in assessing motivations related to palatable food consumption. Indeed, this self-report tool could complement existing measures of compulsive or emotional eating, helping to predict the clinical risk of obesity and supporting the development of customized prevention and treatment strategies against obesity, binge eating, and food addiction.

## Figures and Tables

**Table 1:** Items’ descriptive statistics and confirmatory factor analysis (CFA) results.

	Descriptive Statistics				Properties	CFA	
	M	SD	Sk	K	*r*_it-tot_	λ	*R* ^2^
Social							
Item#3	2.112	0.971	0.695	−0.156	0.694	0.750	0.563
Item#5	1.555	0.801	1.584	2.544	0.550	0.622	0.387
Item#11	1.719	0.867	1.177	1.030	0.665	0.745	0.555
Item#14	1.891	0.994	0.927	−0.051	0.706	0.793	0.630
Item#16	2.524	1.103	0.402	−0.721	0.624	0.689	0.474
Coping							
Item#1	1.802	0.939	1.339	1.561	0.754	0.779	0.607
Item#4	2.123	1.124	0.880	−0.036	0.867	0.921	0.848
Item#6	2.222	1.091	0.860	0.104	0.775	0.832	0.692
Item#15	2.055	1.094	0.974	0.190	0.817	0.865	0.749
Item#17	1.630	0.972	1.627	1.961	0.734	0.756	0.571
Reward enhancement							
Item#7	2.644	1.220	0.333	−0.870	0.738	0.838	0.701
Item#9	1.508	0.840	1.857	3.384	0.516	0.541	0.292
Item#10	1.300	0.679	2.674	7.903	0.363	0.391	0.153
Item#13	2.713	1.195	0.328	−0.854	0.732	0.836	0.698
Item#18	3.112	1.276	−0.060	−1.152	0.638	0.739	0.546
Conformity							
Item#2	1.880	0.903	1.024	0.617	0.274	0.317	0.100
Item#8	1.170	0.555	4.298	21.771	0.600	0.733	0.537
Item#12	1.367	0.700	2.268	5.746	0.636	0.756	0.572
Item#19	1.170	0.486	3.470	14.535	0.625	0.748	0.560
Item#20	1.185	0.527	3.711	17.259	0.641	0.807	0.651

Note: M = mean; SD = standard deviation; Sk = skewness; K = kurtosis; *r*_it-tot_ = item–total correlation; λ = standardized factor loading; *R*^2^ = explained variance.

**Table 2:** Correlation analysis.

		1	2	3	4	5	6	7	8	9	10	11	12	13	14	15
1	PEMS-SM	-														
2	PEMS-CM	0.203 ***	-													
3	PEMS-REM	0.447 ***	0.436 ***	-												
4	PEMS-CM	0.546 ***	0.151 ***	0.129 **	-											
5	mYFAS2.0	−0.004	0.426 ***	0.113 *	0.063	-										
6	BES	0.028	0.587 ***	0.229 ***	0.163 **	0.740 ***	-									
7	MEC10-IT	0.110	0.554 ***	0.248 ***	0.149 *	0.492 ***	0.804 ***	-								
8	TFEQ-CR	0.015	−0.068	−0.065	0.001	0.322 ***	0.402 ***	0.363 ***	-							
9	TFEQ-UE	0.084	0.498 ***	0.343 ***	0.088	0.439 ***	0.656 ***	0.775 ***	−0.018	-						
10	TFEQ-EE	0.122 *	0.772 ***	0.255 ***	0.157 **	0.395 ***	0.624 ***	0.688 ***	−0.038	0.602 ***	-					
11	Rep(Eat)-Q	0.153 ***	0.528 ***	0.282 ***	0.146 **	0.437 ***	0.601 ***	0.599 ***	−0.077	0.574 ***	0.636 ***	-				
12	Rep(Eat)-Q-RE	0.104 *	0.407 ***	0.247 ***	0.097 *	0.273 ***	0.405 ***	0.407 ***	−0.061	0.449 ***	0.508 ***	0.903 ***	-			
13	Rep(Eat)-Q-CG	0.172 ***	0.550 ***	0.266 ***	0.167 ***	0.521 ***	0.690 ***	0.681 ***	−0.079	0.589 **	0.644 **	0.912 ***	0.647 ***	-		
14	BMI	−0.041	0.131 **	−0.037	−0.049	0.389 ***	0.336 ***	0.137 *	−0.071	0.098 *	0.128 *	0.048	0.025	0.062	-	
15	Age	−0.152 **	−0.085 *	−0.134 *	−0.157 **	0.134 *	0.089	−0.073	0.045	−0.144 **	−0.135 **	−0.101 *	−0.092 *	−0.091 *	0.349 ***	-
16	Sex (M/F) ^a^	−0.005	0.180 ***	−0.025	0.064	0.061	0.183 ***	0.089	0.156 **	0.065	0.204 ***	0.092 *	0.012	0.157 ***	−0.361 ***	−0.195 ***

Note: * = *p* < 0.050; ** = *p* < 0.005; *** = *p* < 0.001. ^a^ = adapted for nominal data. PEMS-SM = Palatable Eating Motives Scale-Social motives; PEMS-CM = Palatable Eating Motives Scale-Coping motive; PEMS-REM = Palatable Eating Motives Scale-Reward enhancement motive; PEMS-CM = Palatable Eating Motives Scale-Conformity motive; mYFAS2.0 = Modified Yale Food Addiction Scale 2.0; BES = Binge Eating Scale; MEC10-IT = Measure of Eating Compulsivity—Italian Version; TFEQ-CR = Three Factor Eating Questionnaire-Cognitive Restraint; TFEQ-UE = Three Factor Eating Questionnaire-Uncontrolled Eating; TFEQ-EE = Three Factor Eating Questionnaire-Emotional Eating; Rep(Eat)-Q-RE = Repetitive Eating Questionnaire-Repetitive Eating; Rep(Eat)-Q-CG = Repetitive Eating Questionnaire-Compulsive Grazing.

## Data Availability

Data are available on request due to privacy and ethical restrictions.

## Supplementary Materials

The following supporting information can be downloaded at https://www.mdpi.com/article/10.3390/healthcare12050574/s1, Supplementary Materials S1: The Palatable Eating Motives Scale–Italian version (PEMS-IT).
