# Supplementary material for: Measuring Motivations to Eat Palatable Foods: Adaptation and Psychometric Properties of the Italian Version of the Palatable Eating Motives Scale (PEMS-IT)
Source: Healthcare (Basel). 2024 Feb 29;12(5):574. doi: 10.3390/healthcare12050574 (PMC10930930; doi:10.3390/healthcare12050574)
Supplement: Supplementary file 1 [file healthcare-12-00574-s001.zip › healthcare-2846253-supplementary.pdf]

The Palatable Eating Motives Scale – Italian version (PEMS-IT)

ISTRUZIONI:
Qui sotto è riportata una lista delle ragioni che talvolta spingono le persone a consumare cibi e bevande appetitosi, ad esempio:

- Dolci come cioccolata, ciambelle, biscotti, torte, caramelle, gelato, altri dessert
- Snack salati come patatine, salatini, cracker e noccioline
- Fast food o qualsiasi altro cibo fritto fatto in casa come hamburger, cheeseburger, pizza, pollo fritto e patatine fritte
- Bevande zuccherate come soda, tè dolce, milk-shake e bevande dolci al caffè.

Pensando a tutte le volte che hai consumato questi tipi di cibi o bevande, quanto spesso diresti che li hai mangiati o bevuti per ciascuna delle seguenti ragioni? Segna la risposta che meglio ti descrive **NON ESISTONO RISPOSTE GIUSTE O SBAGLIATE**

1	2	3	4	5
MAI / QUASI MAI	QUALCHE VOLTA	METÀ DELLE VOLTE	IL PIÙ DELLE VOLTE	QUASI SEMPRE / SEMPRE

1	Consumo questi cibi o bevande per dimenticare le mie preoccupazioni	1	2	3	4	5
2	Consumo questi cibi o bevande perché sono invogliato dai miei amici, o dai miei familiari, a mangiarle o berle	1	2	3	4	5
3	Consumo questi cibi o bevande perché mi aiuta a godermi una festa	1	2	3	4	5
4	Consumo questi cibi o bevande perché mi aiuta quando mi sento depresso o nervoso	1	2	3	4	5
5	Consumo questi cibi o bevande per essere socievole	1	2	3	4	5
6	Consumo questi cibi o bevande per tirarmi su di morale quando sono di cattivo umore	1	2	3	4	5
7	Consumo questi cibi o bevande perché mi piace la sensazione.	1	2	3	4	5
8	Consumo questi cibi o bevande per evitare che gli altri mi prendano in giro perché non li mangio o bevo	1	2	3	4	5
9	Consumo questi cibi o bevande perché è stimolante.	1	2	3	4	5
10	Consumo questi cibi o bevande per sentirmi su di giri o provare euforia	1	2	3	4	5
11	Consumo questi cibi o bevande perché rende più divertenti le occasioni di ritrovo sociale	1	2	3	4	5
12	Consumo questi cibi o bevande per sentirmi parte di un gruppo di persone che mi piace	1	2	3	4	5
13	Consumo questi cibi o bevande perché mi danno una sensazione piacevole	1	2	3	4	5
14	Consumo questi cibi o bevande perché mi fanno vivere meglio feste e ricorrenze	1	2	3	4	5
15	Consumo questi cibi o bevande perché mi aiutano a ridurre lo stress	1	2	3	4	5
16	Consumo questi cibi o bevande per celebrare un'occasione speciale con amici o familiari	1	2	3	4	5
17	Consumo questi cibi o bevande per dimenticare i miei problemi	1	2	3	4	5
18	Consumo questi cibi o bevande per il piacere di farlo	1	2	3	4	5
19	Consumo questi cibi o bevande per piacere agli altri	1	2	3	4	5
20	Consumo questi cibi o bevande così da non sentirmi tagliato fuori	1	2	3	4	5
